# Flexible-to-rigid transition is central for substrate transport in the ABC transporter BmrA from *Bacillus subtilis*

**DOI:** 10.1038/s42003-019-0390-x

**Published:** 2019-04-29

**Authors:** Denis Lacabanne, Cédric Orelle, Lauriane Lecoq, Britta Kunert, Claire Chuilon, Thomas Wiegand, Stéphanie Ravaud, Jean-Michel Jault, Beat H. Meier, Anja Böckmann

**Affiliations:** 1 Molecular Microbiology and Structural Biochemistry, UMR 5086 CNRS/Université de Lyon, Labex Ecofect, 7, passage de Vercors, 69367 Lyon, France; 2 Physical Chemistry, ETH Zurich, Vladimir-Prelog-Weg 2, 8093 Zurich, Switzerland

**Keywords:** Bacteria, Solid-state NMR

## Abstract

ATP-binding-cassette (ABC) transporters are molecular pumps that translocate molecules across the cell membrane by switching between inward-facing and outward-facing states. To obtain a detailed understanding of their mechanism remains a challenge to structural biology, as these proteins are notoriously difficult to study at the molecular level in their active, membrane-inserted form. Here we use solid-state NMR to investigate the multidrug ABC transporter BmrA reconstituted in lipids. We identify the chemical-shift differences between the inward-facing, and outward-facing state induced by ATP:Mg^2+^:Vi addition. Analysis of an X-loop mutant, for which we show that ATPase and transport activities are uncoupled, reveals an incomplete transition to the outward-facing state upon ATP:Mg^2+^:Vi addition, notably lacking the decrease in dynamics of a defined set of residues observed in wild-type BmrA. This suggests that this stiffening is required for an efficient transmission of the conformational changes to allow proper transport of substrate by the pump.

## Abstract (web summary)

Lacabanne and Orelle et al. investigate a multidrug ABC transporter BmrA mutant defective in the X-loop, which uncouples ATP hydrolysis from substrate translocation. This study suggests that stiffening of a defined set of amino-acid residues allows the pump to transport substrates properly.

## Introduction

**Fig. 1:**
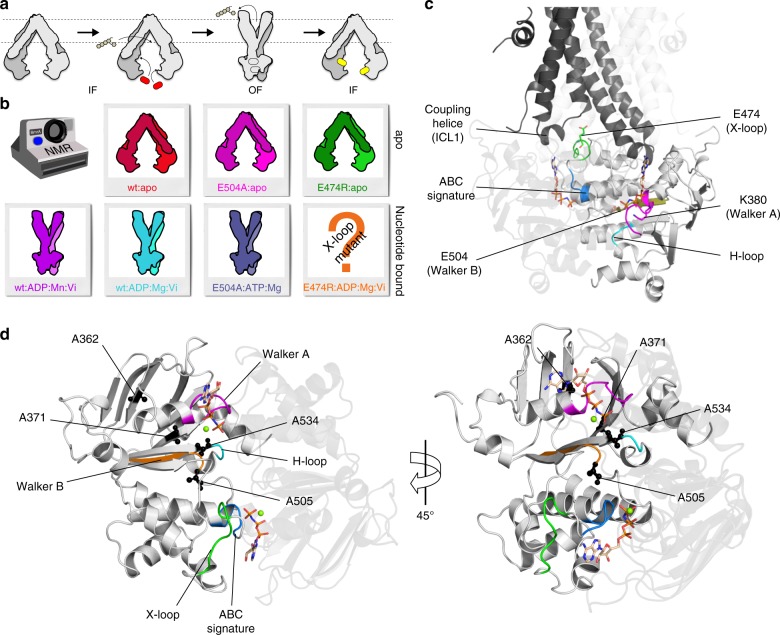
ABC transporter export cycle and samples studied. **a** Putative export cycle of an ABC exporter adapted from Grossmann et al.^26^ and Parcej et al.^27^. A sketched model of the BmrA homodimer is given in gray, ATP is indicated in red, and ADP in yellow. Bound nucleotides in undetermined hydrolysis states are shown in gray. **b** Mimics of different BmrA states investigated, as desribed in the text. **c** BmrA homology model based on Sav1866 (pdb 2hyd^2^), the two mononers are shown in light gray and dark gray colors. Mutated residues are highligthed, with E474 from the X-loop motif (470-TEVG**E**RG-476) in green, residue K380 from the Walker A motif (374-GPSGG**K**T-381) in magenta, and residue E504 from Walker B (496-ILMLD**E**-504) in dark yellow. The ABC signature (477-LSGGQ-483, in blue) and the H-loop (532-AHR-536, in cyan) motifs are also presented. **d** View of the NBDs of BmrA modeled on Sav1866 (pdb 2hyd^2^). A362, A371, A505, and A534 (as discussed in the text) are shown in black spheres, with the Mg^2+^ ions represented in light green sphere

ATP-binding cassette (ABC) exporters can translocate a variety of molecules across the cell membrane via an ATP fueled engine. Notably exporters can be involved in multidrug resistance phenotypes, thereby participating to antimicrobial resistance in yeasts and bacteria, or drug resistance in human anti-cancer chemotherapies. Energy for transport in these proteins is provided through a catalytic cycle, occurring in the nucleotide-binding domains (NBDs), including ATP binding, hydrolysis, and ADP/inorganic phosphate (Pi) release. ABC transporters generally function via an alternating access mechanism in which the catalytic cycle in the NBDs is coupled to the re-orientation of the transmembrane domains (TMDs). The conformational changes, transmitted from the NBDs to the TMDs, reorient the drug-binding site from an inward-facing (IF) to an outward-facing (OF) conformation, resulting in the translocation of drugs across the membrane^1–4^ (see Fig. 1a for a schematic representation).

In exporters, the mode of transmission of the conformational changes between the NBDs and the TMDs remains unclear, although it presumably involves regions of the NBDs that contact the coupling helices of the TMDs^2,5–7^. The transition from the inward-facing to the outward-facing state represents a key event in the pump catalytic cycle and it may coincide with substrate export, although this point is debated for one transporter^8^. It has been described to be either coupled to ATP binding^9,10^ or ATP hydrolysis^8,11,12^. Two main models have been proposed: the ATP-switch model^13^ (or the related processive clamp model^14^) in which ATP binding brings the two NBDs into contact and the constant contact model^15^, where the NBDs remain in contact and ATP is hydrolyzed alternatively in one or the other^16^. Today, no experimental technique is available which allows following in real time the conformational states induced by drug binding and ATP-hydrolysis events. Still, a tool-box of ATP analogs and mutant constructs has been devised to allow approximate instant snapshots, which capture trapped states, designed to be as close to the actual states of the transport cycle as possible^9^. In this context, studying the protein in membranes is a clear asset as it allows to better mimic functional states^17^.

We here use the homodimeric multidrug ABC exporter BmrA from *B. subtilis* (130 kDa), which has been identified in the *B. subtilis* genome by homology with the human P-glycoprotein^18^. BmrA is able to transport multiple substrates, including Hoechst 33342 and doxorubicin^19^, and the antibiotic cervimycin C^20^. While high-resolution 3D structures of BmrA remain to be determined, different conformational states have been characterized for the apo and ADP:Mg:Vi-trapped forms using biochemical studies^21,22^, electron microscopy^23^, and EPR spectroscopy^24^. We here investigate, using solid-state NMR, different conformational states of BmrA reconstituted in *B. subtilis* lipids. NMR chemical shifts and intensities provide a specific identity or fingerprint for a given protein state, and changes therein are highly sensitive probes for conformational and dynamic changes. The states investigated are shown in Fig. 1b. (Please note that we use hereafter Mg and Mn as short forms for Mg^2+^, the natural cofactor that binds in the NBD, and Mn^2+^, a paramagnetic replacement which typically preserves the functionality of the transporter^24^.) We first identified the NMR fingerprints of the wt:apo (IF) state in red, to use them together with the spectra previously recorded on the wt:ADP:Mg:Vi (OF) (in cyan) and wt:ADP:Mn:Vi (OF) (in purple) states^24^ as reference and basis to identify peaks close to the nucleotide-binding site, or remote from it, via paramagnetic relaxation enhancements (PREs). Wt:ADP:Mg:Vi (OF) mimics the ATP-hydrolysis transition state, and wt:ADP:Mn:Vi (OF) is its equivalent paramagnetic state. We then compared them with two mutant proteins: the non-hydrolytic mutant E504A and the X-loop mutant E474R, with the position of the mutations highlighted on the BmrA homology model shown in Fig. 1c. We analyzed the conformational and dynamic features of the ATP-trapped prehydrolytic state E504A:ATP:Mg (OF)^25^ (in blue, and E504A:apo for reference in pink), and of the E474R:ADP:Mg:Vi X-loop mutant, where the conserved residue E474 is replaced by an arginine (in orange), and E474R:apo (in green).

These NMR analyses allowed us to show that ATP binding is sufficient for BmrA in membranes for transition to the outward-facing state, and no hydrolysis is needed at this stage. We established that the X-loop mutant E474R showed uncoupled transport and ATPase activities, and determined that stiffening of a subset of residues seems central in connecting these two processes in order to transform the chemical energy from ATP hydrolysis into mechanical energy to achieve transport.

## Results

**Fig. 2:**
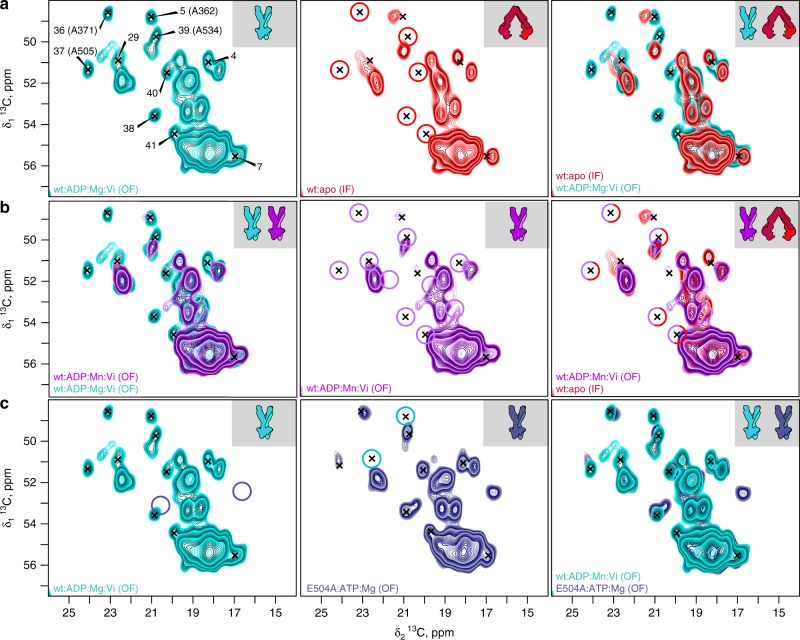
Spectral differences between investigated protein states (for additional regions see Supplementary Fig. 3A, 4A and 6B). **a** wt:apo (IF) and wt:ADP:Mg:Vi (OF) states display different spectral fingerprints. 2D DARR extracts showing Ala region peaks for wt:apo (IF) (red) and wt:ADP:Mg:Vi (OF) (cyan, spectrum from Wiegand et al.^24^) states. Analyzed peaks are marked in all panels by crosses. Signals only observed in the wt:ADP:Mg:Vi (OF) state are highlighted by red circles (middle panel). **b** PRE attenuates signals near the Mn binding site. Comparison between spectra of wt:ADP:Mg:Vi and wt:ADP:Mn:Vi (OF) (left and middle panels, spectrum from Wiegand et al.^24^). Peaks erased by PRE are highlighted by purple circles (middle panel). Comparison of wt:apo (IF) and wt:ADP:Mn:Vi (OF) (right panel), red/purple circles highlight the signals observed in neither state. **c** Mimics of the prehydrolytic and transition states display similar conformations. Same spectral regions comparing the E504A:ATP:Mg (OF) state (dark blue) to the wt:ADP:Mg:Vi (OF) state in cyan; the spectra are, with exception of the modifications due to the mutation highlighted by circles, virtually identical

All spectra shown in the following have been recorded on selectively unlabeled [^12^C-^14^N-LVIKHP]-^13^C-^15^N protein^28^ in order to decongest the spectra. Figure 2 shows extracts of the Ala region of the 2D ^13^C–^13^C DARR correlation spectra. Full spectra were analyzed and are shown in the Supplementary Figures indicated. Coloring of the spectra is according to icons in Fig. 1b. Individual serial numbers are assigned to all peaks which were analyzed, in addition to individual assignments where available^24^. More complete sequential assignments remain largely out of reach for this 589-residue protein today (as discussed in detail in Supplementary Fig. 1 caption).

### The transporter stiffens upon transition to the outward-facing state

In the apo state, BmrA in lipids adopts an inward-facing conformation^23^, which, under the conditions used, forms mesoscopic ring-shaped particles that assemble further into tubes^29–31^. We have recorded spectra of this wt:apo (IF) state, and compared them with spectra previously recorded on the protein in presence of vanadate, ATP, and magnesium^24^, which traps the transporter in an outward-facing conformation mimicking the transition state. We verified that the wt:ADP:Mg:Vi form was trapped in the outward-facing state, by addition of ATP:Mg:Vi and limited proteolysis using trypsin (Supplementary Fig. 2). Indeed, only the outward-facing state resists to trypsin digestion^22^.

Extracts of the alanine region of the spectra of the two samples are shown in Fig. 2 in red for the wt:apo (IF) state, and in cyan for the wt:ADP:Mg:Vi (OF) state (for larger extracts see Supplementary Fig. 3A). The overlay reveals several peaks shifting to different resonance frequencies, which we refer to as chemical-shift perturbations (CSPs)^32^, and which vary here between 0.15 ppm, a threshold which corresponds to more than half of the NMR signal linewidth, and 1 ppm (Supplementary Fig. 3B). CSPs alone can, however, not account for all differences in the spectra, since one can notice, also in Supplementary Fig. 3, that the spectra of the wt:ADP:Mg:Vi (OF) state feature a large number of additional cross peaks, localized by red circles in the spectrum of the wt:apo (IF) state in Fig. 2a. In solid-state NMR measurements, residues showing dynamics with sizeable amplitude on a microsecond time scale are strongly broadened, which can attenuate their peak amplitude beyond detection. The broadening is due to interference of the protein dynamics with the heteronuclear decoupling and/or magic-angle spinning. Thus, additional signals appearing in protein spectra indicate a decrease in the flexibility of the corresponding residues (if they are not observed in solution-type INEPT spectra, which is not the case here). Further characterization of the dynamics is difficult, as the peaks are not even observed at the lowest temperatures where resolution is, at least partially, retained (here −10 °C). At even lower temperatures, resolution deteriorates severely, as generally observed in solid-state NMR of proteins^33^. Four of the six Ala resonances not observed in the wt:apo (IF) state are clearly located in regions indicative for β-sheets or turns, remote from the region around 55 and 18 ppm, where the large signals from α-helical Ala residues concentrate, and three of them could indeed be assigned to residues in β-sheets (A371, A505, A534)^24^ (Supplementary Fig. 1). These alanines belong to the NBD of BmrA and they are highlighted in a homology model built from Sav1866 in Fig. 1d. Two more peaks are located in turn regions (#38, 41). While the additional resonances in wt:ADP:Mn:Vi (OF) could in principle originate also from peak doubling due to occupation of only a single ATP:Mg-binding site, we can exclude this due to the large excess of ATP:Mg (×3000) added to induce the outward-facing. Moreover, EPR distance measurements on the Mn^2+^-substituted protein revealed a distance of 1.9 nm between the two Mn^2+^ atoms, as expected from the homology model of the outward-facing state. This shows that both sites were indeed occupied by nucleotides-Mn in wt:ADP:Mn:Vi (OF)^24^. Eight other signals only visible in the spectra of the wt:ADP:Mn:Vi (OF) state can be identified in the remaining regions of the spectrum (peaks in Supplementary Fig. 3A, gray bars in Supplementary Fig. 3B).

### The rigid residues in the outward-facing state are located in the NBDs

Three Ala residues which are only observed in wt:ADP:Mg:Vi (OF) state could be assigned to individual amino acids and are located within the NBD, namely A371, A505, and A534. In order to see whether this localization can be generalized to the other eleven residues only observed in wt:ADP:Mg:Vi (OF), we used paramagnetic relaxation enhancements to assign the signals to two different regions of the protein, near the ATP-binding site (mainly NBD), and remote from it, including, but not fully restricted to, the TMD. Indeed, Mn^2+^ induces PREs that strongly attenuate resonances from ^13^C spins located within a radius of about 15 Å around the metal ion in 2D DARR spectra^24^, which concerns exclusively resonances in the NBD, as shown on a BmrA homology model from Sav1866^2^ in Supplementary Fig. 4C. The resonances which are far from the Mn^2+^ binding site, notably from the TMD, will not be attenuated by PREs and remain visible in the spectrum. It should be noted that when the Mg^2+^ is replaced by Mn^2+^, the protein retains full transport activity^24^.

We thus compared the spectra of wt:ADP:Mg:Vi (OF) and wt:ADP:Mn:Vi (OF)^24^ with those of the wt:apo (IF) state to identify which of the analyzed residues are in the vicinity of the Mn^2+^ metal ion. In the left panel of Fig. 2b, the spectra of wt:ADP:Mg:Vi (OF) and wt:ADP:Mn:Vi (OF) states are superimposed (larger extracts are shown in Supplementary Fig. 4A). Signals clearly observed only in wt:ADP:Mg:Vi (OF) are highlighted, in the middle panel, by purple circles. Interestingly, when overlaying spectra recorded on wt:ADP:Mn:Vi with those recorded on the wt:apo (IF) state (right panel), one can see that five out of six Ala residues are invisible in both spectra when compared with wt:ADP:Mg:Vi (OF) (dashed red/purple circles). The other alanine (#40) is strongly attenuated in the wt:ADP:Mn:Vi (OF) spectrum. A similar conclusion holds for the remaining analyzed signals (Supplementary Fig. 4B), where only two (#40 and 45) show weak signals, and the others are not observed. The Cα atoms of the three assigned Ala residues (A371, A505, A534) are indeed located, in the BmrA homology model, at 12.6, 9.9, and 8.0 Å from the metal center, close to the Walker A motif (375-GPSGGKT-381) which wraps around the β- and γ-phosphate of ATP, the catalytic glutamate E504 located in the Walker B motif, and the H-loop motif which forms a chemical hinge between the catalytic glutamate and the γ-phosphate of ATP (Fig. 1c).

Because of the similarity between the wt:ADP:Mn:Vi (OF) and the wt:apo (IF) spectra, one could argue that the protein did not bind the paramagnetic ion, and remained in wt:apo (IF). The observation by EPR of two Mn^2+^ ions per dimer in this preparation provides, however, evidence that the ions are bound, and that the protein is indeed in wt:ADP:Mn:Vi (OF)^24^. Further evidence for successful binding stems from a comparison of spectral fingerprints presented in Supplementary Fig. 5, which show that NMR signals which are not attenuated in the wt:ADP:Mn:Vi (OF) spectrum indeed do show the typical chemical-shift fingerprint observed for wt:ADP:Mg:Vi (OF) and not the one corresponding to wt:apo (IF). We can thus conclude that the residues that show dynamic behavior in wt:apo (IF) and become rigid in wt:ADP:Mg:Vi (OF), i.e., the residues which are stiffening when the transporter is in the trapped transition state as opposed to the apo state, are located in the NBDs.

### ATP hydrolysis is not required to induce the outward-facing state

With the catalytic mutation E504A adjacent to the Walker-B motif, BmrA binds, but does not hydrolyze ATP^25^. The mutant has been shown to still undergo, in analogy to wild-type BmrA, a conformational change on incubation with ATP:Mg, destroying the typical mesoscopic ring structures BmrA can form in the wt:apo (IF) state^21^. While wt:ADP:Mg:Vi (OF) can be considered as an ATP hydrolysis transition-state mimic, E504A:ATP:Mg (OF) mimics a prehydrolytic ATP-trapped state^25^. The comparison between the spectra of wt:apo and E504A:apo both in inward-facing state (Supplementary Fig. 6A) reveals largely similar spectra. The two proteins in the presence of nucleotides also look highly similar, as shown in Fig. 2c (for larger extract see Supplementary Fig. 6B, and for numerical analysis Supplementary Fig. 6C), where the few differences, likely due to the mutation, are indicated by circles. E504A:ATP:Mg (OF) thus clearly shows the fingerprint of wt:ADP:Mg:Vi (OF), which allows us to conclude that no ATP hydrolysis is needed to induce the outward-facing state of BmrA in lipids, and that for the mimics used here, the conformation of the outward-facing transition state is, on a molecular level, highly similar to the outward-facing prehydrolytic state in BmrA. This is consistent with the results of Oldham and Chen on the maltose transporter, who showed that, there as well, these two states are essentially similar and could be discernable only in few residues at the level of side-chain in the catalytic site^34–36^.

### BmrA E474R uncouples ATPase and transport activities

**Fig. 3:**
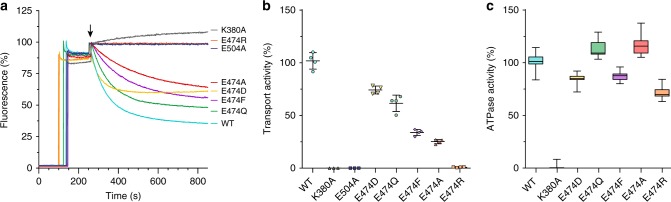
Transport and ATPase activity of BmrA mutants. **a** ATP-dependent transport of doxorubicin measured using inverted *E. coli* membrane vesicles. After addition of 10 µM doxorubicin (120 s), 2 mM of Mg^2+^ were added to initiate transport (indicated by black arrow). **b** Transport activities derived from initial rate fluorescence decays measured for BmrA wild-type and mutant forms, normalized to the rate measured in the wild-type form. **c** ATPase activity of BmrA wild-type and mutant forms purified and reconstituted in *E. coli* lipids with a lipid/protein ratio of 20. Each experiment was conducted three times and error bars indicate the standard deviation

In ABC exporters, the X-loop has been proposed to play a pivotal role as a connecting part between the NBD and the TMD^2,7,37,38^. It contains a strictly conserved glutamate residue (sequence LPNQFDTEVG**E**^**474**^RGIML in BmrA). We prepared the X-loop mutants E474D, E474Q, E474F, E474A, and E474R (see Fig. 1c) and studied their doxorubicin and Hoechst 33342 transport activities using inverted *E. coli* membrane vesicles containing the overexpressed proteins^19^. All mutants were expressed and incorporated at similar levels in the membrane fractions (Supplementary Fig. 7). We first compared the transport activity of each mutant with the wild-type, and also the Walker-A (K380A) and catalytic glutamate (E504A) inactive mutants^21^. The doxorubicin transport profiles as measured by real-time fluorescence spectroscopy are displayed in Fig. 3a (for Hoechst 33342 see Supplementary Fig. 8), and the deduced initial rates are shown in Fig. 3b. It can be clearly seen that the E474R mutant features no transport activity, as also the controls (K380A and E504A). Mutants E474D, E474Q, E474F, and E474A display about 75, 60, 35, and 25%, respectively, of the wild-type protein transport activity. This highlights the deleterious effect of X-loop mutations on transport activity, as also reported for Tap1/Tap2^7^. In order to avoid the high background of ATPase activity due to the presence of numerous ATPases in inverted membrane vesicles, we purified each protein and studied the ATPase activity after reconstitution into proteoliposomes (Fig. 3c). Importantly, the difference between wild-type and mutant proteins is rather small, with the E474R mutant still showing ∼70% activity of the wild-type BmrA ATPase activity. The control (K380A) shows no ATPase activity at all, as observed before^21^. This allowed us to conclude that the mutation hardly impacts the motor domain, which remains almost fully functional in E474R BmrA.

In order to verify that impaired transport is not due to altered drug-binding affinity in the mutant, we monitored binding of doxorubicin and Hoechst 33342 to wild-type and E474R BmrA using intrinsic fluorescence quenching. *K*_D_ values (with standard deviations) of 17 ± 10 μM and 14 ± 8 μM were obtained for doxorubicin binding to wild-type and mutant proteins, respectively, and 12 ± 3 μM and 10 ± 5 μM for Hoechst 33342, confirming that both proteins bind these compounds with similar affinity. It has been shown that the ATPase activity of BmrA is slightly stimulated by the presence of drugs, e.g., reserpine^19^. We measured drug-stimulated ATPase activities of both wild-type BmrA and the E474R mutant, which are both increased by 15% in presence of 3.5 μM reserpine (Supplementary Fig. 9). The observation that the E474R mutant is able to hydrolyze ATP and to bind drugs, but fails to transport them, points to an impaired transmission between the engine (NBDs) and the TMDs in this mutant.

### BmrA E474R makes an incomplete switch to the outward-facing state

In order to investigate the structural basis of the impaired transport activity of the E474R mutant, we recorded spectra of E474R:apo (IF) and E474R:ADP:Mg:Vi (we verified that the ATPase activity of E474R is indeed sensitive to Vi, with an IC_50_ of 50 µM vs 10 µM for the wt; Supplementary Fig. 10). A comparison of E474R:apo (IF) with wt:apo (IF) (Supplementary Fig. 11) shows that both spectra are highly similar.

**Fig. 4:**
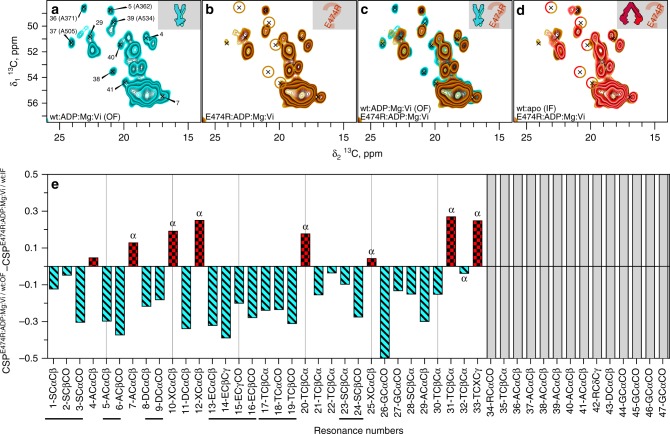
The E474R:ADP:Mg:Vi mutant shares features with both the wt:apo (IF) and wt:ADP:Mg:Vi (OF). **a** extract of 2D DARR spectra of the wt:ADP:Mg:Vi (OF) (for larger extracts see Supplementary Fig. 12). **b** E474R:ADP:Mg:Vi, with brown circles representing peaks which are absent when compared with wt:ADP:Mg:Vi (OF) (Fig. 4a). **c** Overlay of the spectra shown in (**a**) and (**b**). **d** Overlay of the extracts shown in (**b**) with the wild-type protein inward-facing state. Brown-red circles highlight the signals which are only observed in wt:ADP:Mg:Vi (OF), as shown in (**a**). **e** Differences between the CSP^E474R:ADP:Mg:Vi/wt:ADP:Mg:Vi (OF)^ and the CSP^E474R:ADP:Mg:Vi/wt:apo (IF)^. Red positive bars indicate that the CSP^E474R:ADP:Mg:Vi/wt:apo (IF)^ is smaller than the CSP ^E474R:ADP:Mg:Vi/wt:ADP:Mg:Vi (OF)^, and negative cyan bars indicate that the CSP^E474R:ADP:Mg:Vi/wt:ADP:Mg:Vi (OF)^ is smaller than the CSP CSP^E474R:ADP:Mg:Vi/wt:apo (IF)^. “α” indicates that the peak is located in a region where α-helical chemical shifts are located^39^

More interesting is the comparison of the E474R:ADP:Mg:Vi to wt:ADP:Mg:Vi (OF) and wt:apo (IF), as shown in Fig. 4a–d for the Ala region (and in Supplementary Fig. 12 for larger spectral extracts). One can see that several residues observed in the spectra of wt:ADP:Mg:Vi (OF) (Fig. 4a) are actually not observed in the E474R:ADP:Mg:Vi (orange circles in Fig. 4b). Interestingly, these signals coincide largely with those not observed in the wt:apo (IF), as can be seen in the overlay in Fig. 4d, highlighted by the coinciding red/orange circles. For peak #37 (A505), which corresponds to the neighboring residue to the catalytic glutamate (E504), the signal is still observable, but strongly attenuated (factor of 0.4). With respect to dynamics, a similar state to wt:apo (IF) is thus observed for E474R:ADP:Mg:Vi, also indicated by the gray bars in Fig. 4e.

The situation is different with respect to chemical shifts and thus conformation. Figure 4e displays the differences, for peaks 1–33, between the wt:apo (IF)/E474R:ADP:Mg:Vi and the wt:ADP:Mg:Vi (OF)/E474R:ADP:Mg:Vi CSPs as a measure for the similarity of the mutant conformation to either inward-facing or outward-facing states. Negative cyan bars indicate that the peak is closer to wt:ADP:Mg:Vi (OF), and positive red bars that the peak is closer to wt:apo (IF). Twenty-five out of 33 analyzed peaks show negative values, indicative for higher similarity with wt:ADP:Mg:Vi (OF). Interestingly, the chemical shifts of all these peaks point to β-strand secondary structures^39^, exclusively found in the NBDs. This means that at least the β-sheet parts of the NBDs are largely in a conformation similar to wt:ADP:Mg:Vi (OF). Out of the eight peaks with positive values, closer to wt:apo (IF), seven are found in spectral regions rather corresponding to α-helical secondary structure, as indicated by the α above the bars. Out of these seven peaks, only one (#12) is fully attenuated by the presence of Mn (Supplementary Fig. 4B). The others are thus located remotely from the Mn binding site, toward the interface with, or inside the TMDs. The analysis thus points, with respect to conformation, to a mixed state, with the majority of β-strand residues close to wt:ADP:Mg:Vi (OF), and α-helical residues, mainly distant to the active site, close to wt:apo (IF).

This strongly suggests that the state induced in the E474R mutant by the presence of ADP:Mg:Vi can be characterized as an incomplete outward-facing state, in which the NBDs are engaged, thereby trapping the ADP:Mg:Vi at their interface, while remote regions, including the TMDs remain essentially in an inward-facing conformation or an occluded state as observed with McjD^40^. These results point, together with the persistent dynamics (i.e., absence of stiffening), to a rationale for the lack of export activity observed in the mutant.

## Discussion

**Fig. 5:**
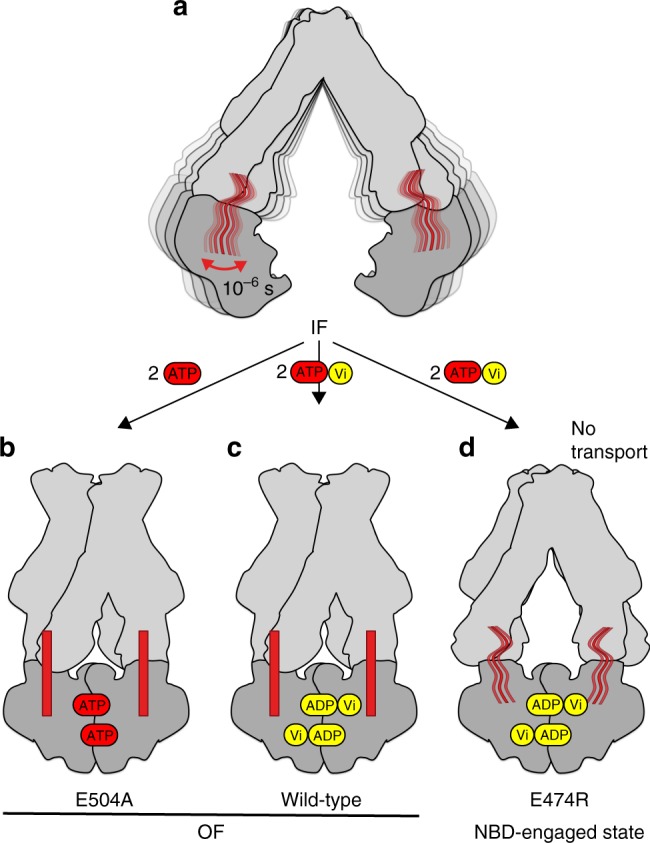
Summary of our findings (highlighted in red). **a** BmrA in its inward-facing state with a subset of residues showing dynamics on the microsecond time scale, which are mainly located within 15 Å of the ATP:Mg-binding site. **b**, **c** BmrA in its outward-facing state, in which a set of residues stiffens, and conformational changes occur around the ATP-binding site, but also remotely in an allosteric manner to allow drug transport. **d** In the presence of ATP:Mg:Vi, the E474R mutant makes an incomplete conformational and dynamic transition, with the NBDs engaged in and ADP:Mg:Vi bound state, but with residues remaining flexible, and only partially observed CSPs

In this work, we have analyzed key states in the transport cycle of an ABC multidrug exporter. We have shown, using the E504A mutant of BmrA, that the inward-facing state (Fig. 5a) can switch to the outward-facing state without ATP hydrolysis, as recapitulated in the cartoon in Fig. 5b. If substrate release is linked in BmrA to the outward-facing transition (which remains to be demonstrated), this would mean that drug release takes place without hydrolysis, as recently proposed for MRP1^41^ and P-glycoprotein transporters^42^. We could show, at atomic level and for the membrane-inserted protein, that the conformation of the prehydrolytic state of BmrA is very similar to that of the transition state (Fig. 5c), as their spectra are, within the technical limits (i.e., linewidth, spectral overlap, signal-to-noise ratio), virtually the same.

It has been discussed in the literature whether in ABC transporters, ATP binding occurs in a symmetric way with both sites occupied^15,43^. In BmrA, we can identify symmetry, through the absence of ^13^C peak doubling, which would support the ATP-switch model^13,44^ involving symmetric nucleotide binding.

Importantly, not only conformational differences, but also differences in dynamics occur between the inward-facing and outward-facing states of BmrA, indicated by the concomitant stiffening of a set of residues in the NBDs. Dynamic behavior in ABC exporters has been identified by different techniques, such as EPR on MsbA^45,46^, P-gp^47^, LmrA^48^, BmrCD^11^, and TM287/288^9^, cryo-electron microscopy for CFTR^49^, ABCG2^50^, MRP1^41^, P-glycroprotein transporters^42^, MsbA^51^, and solid-state NMR for LmrA^52^ or even BmrA by H/D exchange coupled to mass spectrometry^22^. The dynamics we observe here for a set of residues is not necessarily related with these observations from other techniques. In EPR studies, flexibility of the NBDs is revealed through the presence of a variety, coexisting states, which are typically frozen out, and whose presence leads to the detection of an average distance distributions of varying amplitude between NBDs. In cryo-electron microscopy, flexibility in certain transporters is revealed by entire loops or other parts of the protein showing density which is too diffuse to be modeled accurately. In contrast, our observations here by solid-state NMR, like those reported in a parallel study on the DnaB helicase^53^, point to single residues which, depending on the state of the protein, stiffen or become flexible on the time scale of the micro- to milliseconds. These dynamics localize to a restricted set of residues, but clearly do not concern the entire NBD (as indeed there are signals present in the wt:apo state, which disappearance on addition of Mn identifies them as located in the NBDs). The peaks that show up only in the wild-type (or E504A) protein nucleotide-bound states thus represent a subset of amino acids only. These residues become rigid when binding of ATP:Mg:Vi induces  re-orientation of the drug-binding site to the outward-facing state, but do not do so in the E474R mutant protein, where no transport activity is observed. The X-loop has been described as a mediating element connecting the conformational changes caused by ATP hydrolysis in the NBDs to those in the TMDs. In the E474R mutant, the observation of a closed NBD dimer but absence of transport could thus be explained by a loss of coupling through the “coupling helices”. This loss of coupling was also suggested for the conserved Gln of the ABC signature in MDR3/ABCB4^37^. The stiffening of the dynamic residues on ADP:Mg:Vi binding to the wild-type protein, not observed in the E474R mutant, may indicate that a larger set of residues, including from the NBD β-strands, play a role in this transmission. Thus, the loss of dynamics may be required to transmit conformational change from the NBDs to the cytoplasmic ends of the TMDs. This does not only involve the coupling helices, but more likely a larger network of atoms, resulting eventually in opening the drug-binding pocket toward the outside thereby achieving transport. ATP is hydrolyzed by the E474R mutant protein, which means that the engine providing energy is intact. Still, this energy cannot be transformed into mechanical energy where required (Fig. 5d). To obtain a residue-specific view on which ones take part in this dynamic change, more complete sequential resonance assignments of the protein resonances will be needed. This might get into reach with new NMR developments, as proton-detection techniques, as well as the advent of higher magnetic fields delivering the critical sensitivity and resolution for the site-specific study of such large proteins as the one studied here.

## Methods

### NMR sample preparation

The production, purification and reconstitution of BmrA was done as described previously^30,31^. Selectively labeled BmrA was produced in M9-medium supplemented with natural abundance amino acids (see below) in presence of ^13^C-glucose (2 g L^−1^) and ^15^N-ammonium chloride (2 g L^−1^) as sole sources of carbon-13 and nitrogen-15. Pre-cultures, (i) 50 mL at 37 °C then (ii) 150 mL at 25 °C inoculated with (i), were realized before the main culture. Eight hundred and fifty milliliters of M9-medium containing natural abundant Leu (0.23 g L^−1^), Val (0.23 g L^−1^), Ile (0.23 g L^−1^), Lys (0.40 g L^−1^), His (0.40 g L^−1^), and Pro (0.10 g L^−1^) was inoculated with the 150-mL pre-culture at 25 °C. Induction was performed with 0.7 mM IPTG, OD_600nm_ between 0.6 and 0.7. The cells were harvested by centrifugation (6000 × *g*, 15 min) when the stationary phase was reached. The cells were lysed by high pressure using a Microfluidics Microfluidizer^®^ in a lysis buffer composed of 50 mM Tris-HCl pH 8.0, 5 mM MgCl_2_, 1 mM DTT, benzonase, and EDTA-free protease inhibitor cocktail. The solution was centrifuged at 15,000 × *g* during 45 min, the pellet was discarded and the supernatant was centrifuged at 200,000 × *g* during 1 h. The sedimented membranes were suspended and collected in 50 mM Tris-HCl pH 8.0, 1 mM EDTA, and 300 mM sucrose. The protein-containing membranes were diluted at 2 mg mL^−1^, solubilized using 1% DDM (m/v) and centrifuged at 100,000 × *g* for 1 h. The supernatant was loaded onto a Ni-NTA column (Qiagen) equilibrated with 50 mM Tris-HCl pH 8.0, 100 mM NaCl, 15% glycerol, 10 mM imidazole, and 0.2% DDM (m/v). The Ni-NTA column was washed using equilibration buffer containing 0.5 M NaCl, then successively 30 mM imidazole, 40 mM imidazole, and 250 mM imidazole for elution. The eluted protein was desalted using PD10 columns (PD10 - GE Healthcare Life Sciences) which were equilibrated with 50 mM Tris-HCl pH 8.0, 100 mM NaCl, 10% glycerol, and 0.2% DDM (m/v). In order to decrease the concentration of DMM from 0.2 to 0.05% (m/v), the protein solution was diluted four times with 50 mM Tris-HCl pH 8.0, 100 mM NaCl, and 5% glycerol. The solution was mixed with a homemade preparation of *B. subtilis* lipids^30,31^ solubilized in Triton X-100 with a molar ratio of 10:1 and incubated for 1 h. *B. subtilis* lipids in natural abundance are extracted from *B. subtilis* cultures and mainly consist in cardiolipin, phosphatidylethanol-amine, phosphatidyl-glycerol, and lysylphosphatidyl-glycerol (12:30:36:22)^43^. The quantity of lipid mix was adjusted at a lipid-to-protein ratio (LPR, in m:m) of 0.5 or 20. The DDM and Triton X-100 were removed using dialysis with Bio-beads (BioRad). The protein solution was dialyzed against 50 mM Tris-HCl pH 8.0, 100 mM NaCl, and 5% glycerol. during 9 days^31^. An excess of Bio-beads was used. This excess correspond to three times the theoretical amount required to absorb the DDM (1 g Bio-beads for 105 mg) and Triton X-100 (1 g Bio-beads for 70 mg).

For the preparation of the BmrA:ADP:Mg:Vi or BmrA:ADP:Mn:Vi complexes, the protein solutions, after 9 days of dialysis, were incubated with 1 mM Na_3_VO_4_, then 10 mM ATP and 10 mM Mg^2+^ or 1 mM Mn^2+^ during 1 h. For the BmrA-E504A:ATP:Mg complex, the solution was incubated with 10 mM ATP and 10 mM Mg^2+^ during 1 h. The protein in lipids was sedimented into the MAS-NMR rotor (1 h at 4 °C with 120,000 × *g*) using home-build tools^54^. For each sample, 4 L of carbon-13, nitrogen-15 culture yielding a total of 40 mg protein were produced.

### Preparation of BmrA mutants

Site-directed mutagenesis of BmrA X-loop mutants was performed using a Quikchange lightning kit provided by Agilent. The design of the primers was done following the instruction of the provider. K380A and E504A mutants were previously prepared^21,25^. All mutants gave similar expression and purification levels. Hundred-percent ATPase activity in Fig. 3c corresponded to ~ 6 μmol min^−1^ mg^−1^.

### Solid-state NMR experiments

NMR spectra of all samples were acquired on a Bruker Avance spectrometer operating at 800 MHz ^1^H frequency using a 3.2 mm triple-resonance E-free probe. The sample temperature was set to 278 K using the water resonance frequency as a reference^31^. The 2D spectra were processed using TOPSPIN 3.5^55^ with a shifted cos^2^ function with SSB=3 and analyzed using CcpNMR software^56^. No custom codes were used. The details of NMR experiments are given in the Supplementary Table 1.

### Drug transport activity of BmrA in presence of Mg^2+^

Drug transport was followed using fluorescence spectroscopy^19^. In a 1 mL cuvette, 100 µg of inverted membrane vesicles from *E. coli* C41(DE3) overexpressing BmrA were incubated in presence of 2 mM ATP, 4 mM phosphoenolpyruvate, and 60 µg mL^−1^ pyruvate kinase. After 1 min incubation at 25 °C, 10 µM doxorubicin were added and the transport reaction was initiated by adding 2 mM MgCl_2_. The excitation and emission wavelengths used were 480 and 590 nm, respectively. Fluorescence measurements were carried out using a Photon Technology International spectro-fluorimeter with an 814 photomultiplier detection system using a 75 W xenon short arc lamp.

### ATPase activity assay in micro-plate

ATPase activity was monitored using an ATP/NADH coupled assay in micro-plates^31^. In total, 0.1 µg of reconstituted BmrA (LPR 20) was mixed in 50 mM HEPES-KOH pH 8, 4 mM phosphoenolpyruvate, 60 µg mL^−1^ pyruvate kinase, 32 µg mL^−1^ lactate dehydrogenase, 10 mM MgCl_2_, and 0.6 mM NADH. The reaction was started by the addition of 5 mM ATP and the decreasing absorbance of NADH was followed at 340 nm during the reaction (spectrophotometer SAFAS FLX-Xenius^®^) during 20 min.

### Fluorescence binding experiments

Hoechst 33342 and doxorubicin binding were assayed by quenching the tryptophan fluorescence using reconstituted BmrA^25^ in 50 mM Hepes pH 8. Excitation wavelength was 295 nm and emission fluorescence was recorded between 310 and 380 nm. Measurements were carried out using a Photon Technology International spectro-fluorimeter with an 814 photomultiplier detection system using a 75 W xenon short arc lamp. All experiments were performed at 25 °C with 0.33 µM BmrA (monomer concentration) in 1 mL cuvette. Excitation wavelength was 295 nm and emission fluorescence was recorded between 310 and 380 nm. Final quenching spectra were obtained after both corrections from spectra obtained with empty liposomes in the same buffer and from the inner-filter effect by using N-acetyl-tryptophanamide (NATA): three cuvettes were prepared to which we successively added ligand concentrations: empty liposomes, empty liposomes + NATA, and proteoliposomes. For each ligand concentration, the ratio F = (Fc/Fc0)_protein_/(Fc/Fc0)_NATA_ was calculated, where Fc is the fluorescence corrected from the buffer containing empty liposomes and Fc0 similarly corrected in absence of ligand. Quenching data were analyzed according to the equation *F* = Fmax − ((Fmax − Fmin)((Et + *L* + Kd) − ((Et + *L* + Kd)^2^–4Et*L*)^0.5^))/2Et, where Et is the enzyme concentration, *L* the ligand concentration, Fmax is the fluorescence at the start of the titration, and Fmin the fluorescence at saturating concentration of ligand.

### Limited proteolysis experiments

BmrA E474R and wild-type in lipids (LPR 0.5) at ~0.8 mg mL^−1^ were incubated in 50 mM Tris-HCl pH 8, 100 mM NaCl, 5% glycerol (v/v), and 0.5 µg mL^−1^ of porcine trypsin (Sigma) with or without 10 mM ATP, 11 mM MgCl_2_, and 1 mM vanadate. In order to stop proteolysis, 0.5% (v/v) of trifluoroacetic acid and 2% (m/v) SDS were added. The sample was immediately frozen in liquid nitrogen.

### BmrA homology model

The BmrA homology model was built starting from the Sav1866 structure (pdb 2hyd^2^).

### Reporting summary

Further information on experimental design is available in the Nature Research Reporting Summary linked to this article.

## Supplementary information

Reporting Summary

Supplementary Information

## Data Availability

The authors declare that all data supporting the findings of this study are available within the article, its Supplementary Information file and from the corresponding author on reasonable request.

## References

[1] Ward A, Reyes CL, Yu J, Roth CB, Chang G (2007). Flexibility in the ABC transporter MsbA: alternating access with a twist. Proc. Natl Acad. Sci. USA.

[2] Dawson RJP, Locher KP (2006). Structure of a bacterial multidrug ABC transporter. Nature.

[3] Seeger MA, van Veen HW (2009). Molecular basis of multidrug transport by ABC transporters. Biochim. Biophys. Acta.

[4] Locher KP (2016). Mechanistic diversity in ATP-binding cassette (ABC) transporters. Nat. Struct. Mol. Biol..

[5] Jones PM, George AM (2002). Mechanism of ABC transporters: a molecular dynamics simulation of a well characterized nucleotide-binding subunit. Proc. Natl Acad. Sci. USA.

[6] Dalmas O (2005). The Q-loop disengages from the first intracellular loop during the catalytic cycle of the multidrug ABC transporter BmrA. J. Biol. Chem..

[7] Oancea G (2009). Structural arrangement of the transmission interface in the antigen ABC transport complex TAP. Proc. Natl Acad. Sci. USA.

[8] Perez C (2015). Structure and mechanism of an active lipid-linked oligosaccharide flippase. Nature.

[9] Timachi MH (2017). Exploring conformational equilibria of a heterodimeric ABC transporter. eLife.

[10] Doshi R (2013). Molecular disruption of the power stroke in the ATP-binding cassette transport protein MsbA. J. Biol. Chem..

[11] Mishra S (2014). Conformational dynamics of the nucleotide binding domains and the power stroke of a heterodimeric ABC transporter. eLife.

[12] Verhalen B (2017). Energy transduction and alternating access of the mammalian ABC transporter P-glycoprotein. Nature.

[13] Higgins CF, Linton KJ (2004). The ATP switch model for ABC transporters. Nat. Struct. Mol. Biol..

[14] Janas E (2003). The ATP hydrolysis cycle of the nucleotide-binding domain of the mitochondrial ATP-binding cassette transporter Mdl1p. J. Biol. Chem..

[15] George AM, Jones PM (2012). Perspectives on the structure–function of ABC transporters: The Switch and Constant Contact Models. Prog. Biophys. Mol. Biol..

[16] Jones PM, George AM (2011). Molecular-dynamics simulations of the ATP/apo state of a multidrug ATP-binding cassette transporter provide a structural and mechanistic basis for the asymmetric occluded state. Biophys. J..

[17] Alvarez FJD (2015). Full engagement of liganded maltose-binding protein stabilizes a semi-open ATP-binding cassette dimer in the maltose transporter. Mol. Microbiol..

[18] Steinfels E (2002). Highly efficient over-production in *E. coli* of YvcC, a multidrug-like ATP-binding cassette transporter from *Bacillus subtilis*. Biochim. Biophys. Acta.

[19] Steinfels E (2004). Characterization of YvcC (BmrA), a multidrug ABC transporter constitutively expressed in *Bacillus subtilis*. Biochemistry.

[20] Krügel H (2010). Cervimycin C resistance in *Bacillus subtilis* is due to a promoter up-mutation and increased mRNA stability of the constitutive ABC-transporter gene *bmrA*. FEMS Microbiol. Lett..

[21] Orelle C (2008). Conformational change induced by ATP binding in the multidrug ATP-binding cassette transporter BmrA. Biochemistry.

[22] Mehmood S, Domene C, Forest E, Jault JM (2012). Dynamics of a bacterial multidrug ABC transporter in the inward- and outward-facing conformations. Proc. Natl Acad. Sci. USA.

[23] Fribourg PF (2014). 3D Cryo-Electron Reconstruction of BmrA, a Bacterial Multidrug ABC Transporter in an Inward-Facing Conformation and in a Lipidic Environment. J. Mol. Biol..

[24] Wiegand T (2017). Solid-state NMR and EPR spectroscopy of Mn(2 +)-substituted ATP-fueled protein engines. Angew. Chem. Int. Ed..

[25] Orelle C, Dalmas O, Gros P, Di Pietro A, Jault JM (2003). The conserved glutamate residue adjacent to the Walker-B motif is the catalytic base for ATP hydrolysis in the ATP-binding cassette transporter BmrA. J. Biol. Chem..

[26] Grossmann N (2014). Mechanistic determinants of the directionality and energetics of active export by a heterodimeric ABC transporter. Nat. Commun..

[27] Parcej D, Tampé R (2010). ABC proteins in antigen translocation and viral inhibition. Nat. Chem. Biol..

[28] Lacabanne D, Meier BH, Böckmann A (2018). Selective labeling and unlabeling strategies in protein solid-state NMR spectroscopy. J. Biomol. NMR.

[29] Chami M (2002). Three-dimensional structure by cryo-electron microscopy of YvcC, an homodimeric ATP-binding cassette transporter from Bacillus subtilis1. J. Mol. Biol..

[30] Kunert B (2014). Efficient and stable reconstitution of the ABC transporter BmrA for solid-state NMR studies. Frontiers Mol Biosc.

[31] Lacabanne D (2017). Gradient reconstitution of membrane proteins for solid-state NMR studies. J. Biomol. NMR.

[32] Williamson MP (2013). Using chemical shift perturbation to characterise ligand binding. Progr NMR Spectr.

[33] Bauer T (2017). Line-broadening in low-temperature solid-state NMR spectra of fibrils. J. Biomol. NMR.

[34] Oldham ML, Khare D, Quiocho FA, Davidson AL, Chen J (2007). Crystal structure of a catalytic intermediate of the maltose transporter. Nature.

[35] Oldham ML, Chen J (2011). Snapshots of the maltose transporter during ATP hydrolysis. Proc. Natl Acad. Sci. USA.

[36] Oldham ML, Chen J (2011). Crystal structure of the maltose transporter in a pretranslocation intermediate state. Science.

[37] Kluth M (2015). A mutation within the extended X loop abolished substrate-induced ATPase activity of the human liver ATP-binding cassette (ABC) transporter MDR3. J. Biol. Chem..

[38] Xu Y, Seelig A, Bernèche S (2017). Unidirectional transport mechanism in an ATP dependent exporter. ACS Cent. Sci..

[39] Wang Y, Jardetzky O (2002). Probability‐based protein secondary structure identification using combined NMR chemical‐shift data. Protein Sci..

[40] Choudhury HG (2014). Structure of an antibacterial peptide ATP-binding cassette transporter in a novel outward occluded state. Proc. Natl Acad. Sci. USA.

[41] Johnson ZL, Chen J (2017). Structural basis of substrate recognition by the multidrug resistance protein MRP1. Cell.

[42] Kim Y, Chen J (2018). Molecular structure of human P-glycoprotein in the ATP-bound, outward-facing conformation. Science.

[43] Aittoniemi J, de Wet H, Ashcroft FM, Sansom MSP (2010). Asymmetric switching in a homodimeric ABC transporter: a simulation study. PLoS Comp. Biol..

[44] Linton KJ, Higgins CF (2007). Structure and function of ABC transporters: the ATP switch provides flexible control. Pflugers Arch..

[45] Zou P, Bortolus M, Mchaourab HS (2009). Conformational cycle of the ABC transporter MsbA in liposomes: detailed analysis using double electron-electron resonance spectroscopy. J. Mol. Biol..

[46] Mittal A, Böhm S, Grütter MG, Bordignon E, Seeger MA (2012). Asymmetry in the homodimeric ABC transporter MsbA recognized by a DARPin. J. Biol. Chem..

[47] Wen PC, Verhalen B, Wilkens S, Mchaourab HS, Tajkhorshid E (2013). On the origin of large flexibility of P-glycoprotein in the inward-facing state. J. Biol. Chem..

[48] Hellmich UA (2012). Probing the ATP hydrolysis cycle of the ABC multidrug transporter LmrA by pulsed EPR spectroscopy. J. Am. Chem. Soc..

[49] Zhang Z, Chen J (2016). Atomic structure of the cystic fibrosis transmembrane conductance regulator. Cell.

[50] Manolaridis I (2018). Cryo-EM structures of a human ABCG2 mutant trapped in ATP-bound and substrate-bound states. Nature.

[51] Mi, W. et al. Structural basis of MsbA-mediated lipopolysaccharide transport. *Nature* 1–19 https://10.1038/nature23649 (2017). 10.1038/nature23649 PMC5759761 28869968

[52] Siarheyeva A (2007). Probing the molecular dynamics of the ABC multidrug transporter LmrA by deuterium solid-state nuclear magnetic resonance. Biochemistry.

[53] Wiegand T (2019). The conformational changes coupling ATP hydrolysis and translocation in a bacterial DnaB helicase. Nat. Commun..

[54] Böckmann A (2009). Characterization of different water pools in solid-state NMR protein samples. J. Biomol. NMR.

[55] Stevens TJ (2011). A software framework for analysing solid-state MAS NMR data. J. Biomol. NMR.

[56] Bruker Biospin TopSpin 3.5—NMR software download https://www.bruker.com/service/support-upgrades/software-downloads/nmr.html

