# Supplementary material for: Flexible-to-rigid transition is central for substrate transport in the ABC transporter BmrA from Bacillus subtilis
Source: Commun Biol. 2019 Apr 29;2:149. doi: 10.1038/s42003-019-0390-x (PMC6488656; doi:10.1038/s42003-019-0390-x)
Supplement: Supplementary file 2 — Supplementary Information [file 42003_2019_390_MOESM2_ESM.pdf]

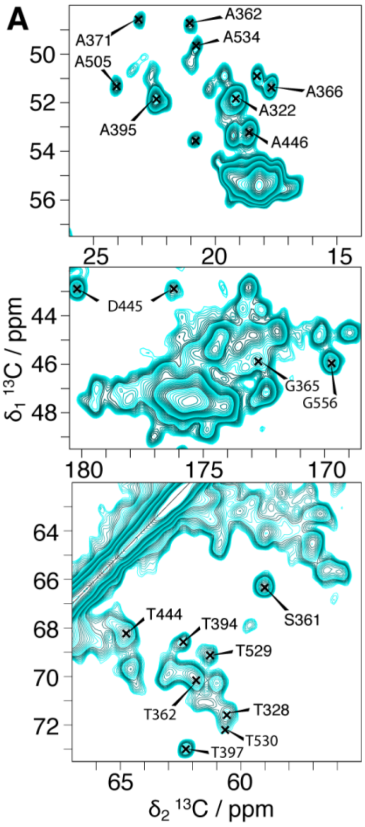

B

Residue	δ(^{13}CO)/ ppm	δ(^{13}CA)/ ppm	δ(^{13}CB)/ ppm
**A322**		51.5	17.9
**T328**		71.7	60.7
**S361**		59.1	66.5
**A362**	175.0	49.0	21.2
**E365**		57.7	30.4
**A366**		51.6	17.9
**G367**	172.9	46.0	
**T394**	174.1	62.5	68.7
**A395**		52.0	22.6
**G396**		44.9	
**T397**	171.7	62.4	73.1
**T444**		64.9	68.3
**D445**	176.3	43.1	57.6
**A446**		53.8	18.8
**A505**		51.5	24.2
**T529**	175.1	61.5	69.2
**T530**	179.0	72.3	60.9
**A534**	176.0	49.9	20.9
**G556**	169.8	46.1	

Supplementary figure 1. Chemical shift values for assigned residues. (*A*) Resonances assignments on the 2D 20 ms DARR spectra on the region of alanine, glycine and threonine/serine. (*B*) ^{13}C chemical-shift values of assigned residues in BmrA:ADP:Mg:Vi. These assignments were obtained from a consistent combination of information from 2D 200 ms DARR spectra recorded on selectively unlabeled samples ([^{12}C-^{14}N-LVIKHP]-^{13}C-^{15}N-BmrA or [^{12}C-^{14}N-LVIRH]-^{13}C-^{15}N), paramagnetic relaxation enhancements[1], as well as sequence and structure information from a homology model created from SAV1866[2]. More extensive sequential assignments remain largely out of reach for this 589 residue protein today, as we observe reduction of the signal to noise (S/N) ratio to 1/3 when compared to similar sized globular proteins such as DnaB (490 residues), which could be partly assigned[1]. One would have expected the presence of lipids (LPR 0.5) to reduce the S/N by only 1/3. Thus, additional factors to sheer size and membrane-inserted state reduce signal to noise further, and we are currently investigating the reasons for this fact, which might lie in the need of higher centrifugal forces for efficient rotor packing, or in the overall dynamics of the protein interfering with NMR parameters. Signal loss by a factor of three increases spectra recording times by a factor of 9, making 3D spectroscopy virtually impossible (about one month per spectrum). Chemical shift predictions like SHIFTX2[3] or SPARTA[4] from the structural model based on SAV1866[2] show, as expected, not enough precision to help with extended assignments.

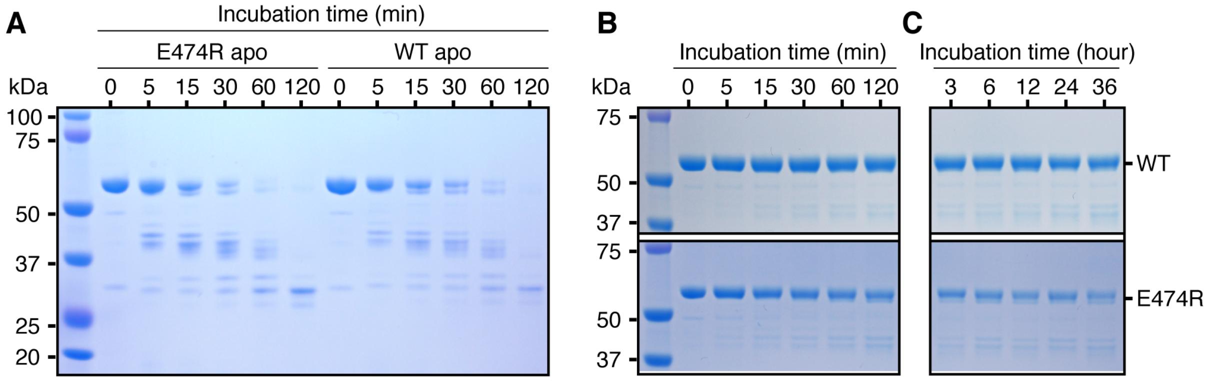

Supplementary figure 2. SDS-PAGE analysis of BmrA purified and reconstituted in lipids submitted to limited proteolysis with trypsin. *(A)* BmrA-E474R and BmrA-wt in the apo conformation after incubation with trypsin for 0, 5, 15, 30, 60 and 120 min (from left to right lanes). *(B)* wt:ATP:Mg:Vi and E474R:ATP:Mg:Vi after incubation with trypsin for 0, 5, 15, 30, 60 and 120 min (from left to right lanes). (*C*) Stability test of the complex wt:ATP:Mg:Vi and E474R:ATP:Mg:Vi over the time. The protein was incubated from 3 to 36 hours in presence of ATP:Mg:Vi. Trypsin was added with the sample during the last two hours in order to test the resistance of the complex.

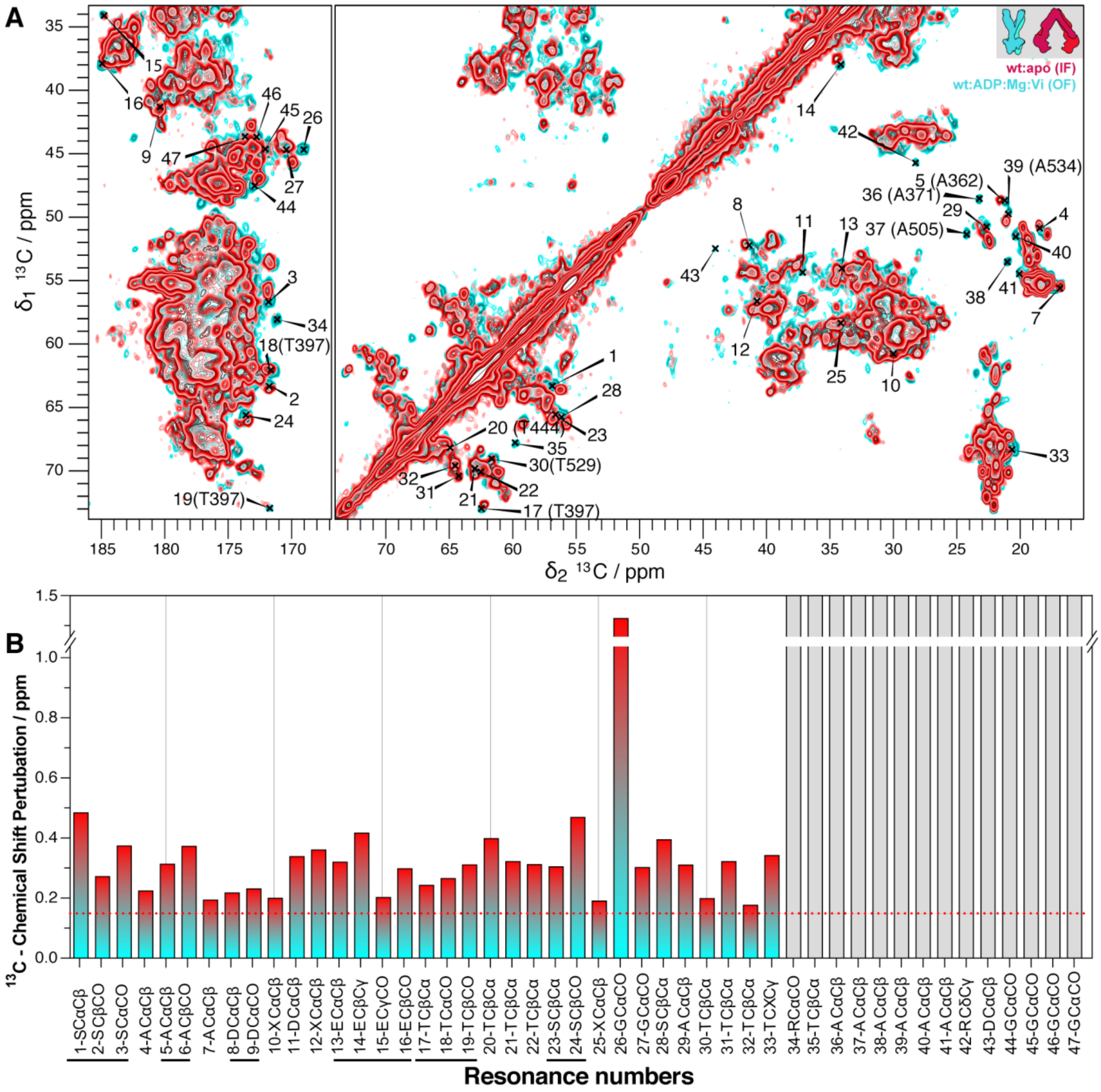

Supplementary figure 3. Extended extracts and peak analysis of wt:apo versus wt:ADP:Mg:Vi spectra. *(A)* Aliphatic and carbonyl region of 2D ^{13}C-^{13}C 20 ms DARR spectra of IF (red) and OF (cyan) states. (*B*) Chemical-shift perturbations (CSPs) from 47 peaks between IF and OF states were calculated using $[0.5(C\alpha_{IF} - C\alpha_{OF})^2 + 0.5(C\beta_{IF} - C\beta_{OF})^2]^{0.5}$ in accordance with Williamson *et al.* [5]. Peaks only observed in the OF state are represented by grey bars. The red dashed line indicates the limit above which we consider the effects to be relevant (CSP higher than 0.15 ppm).

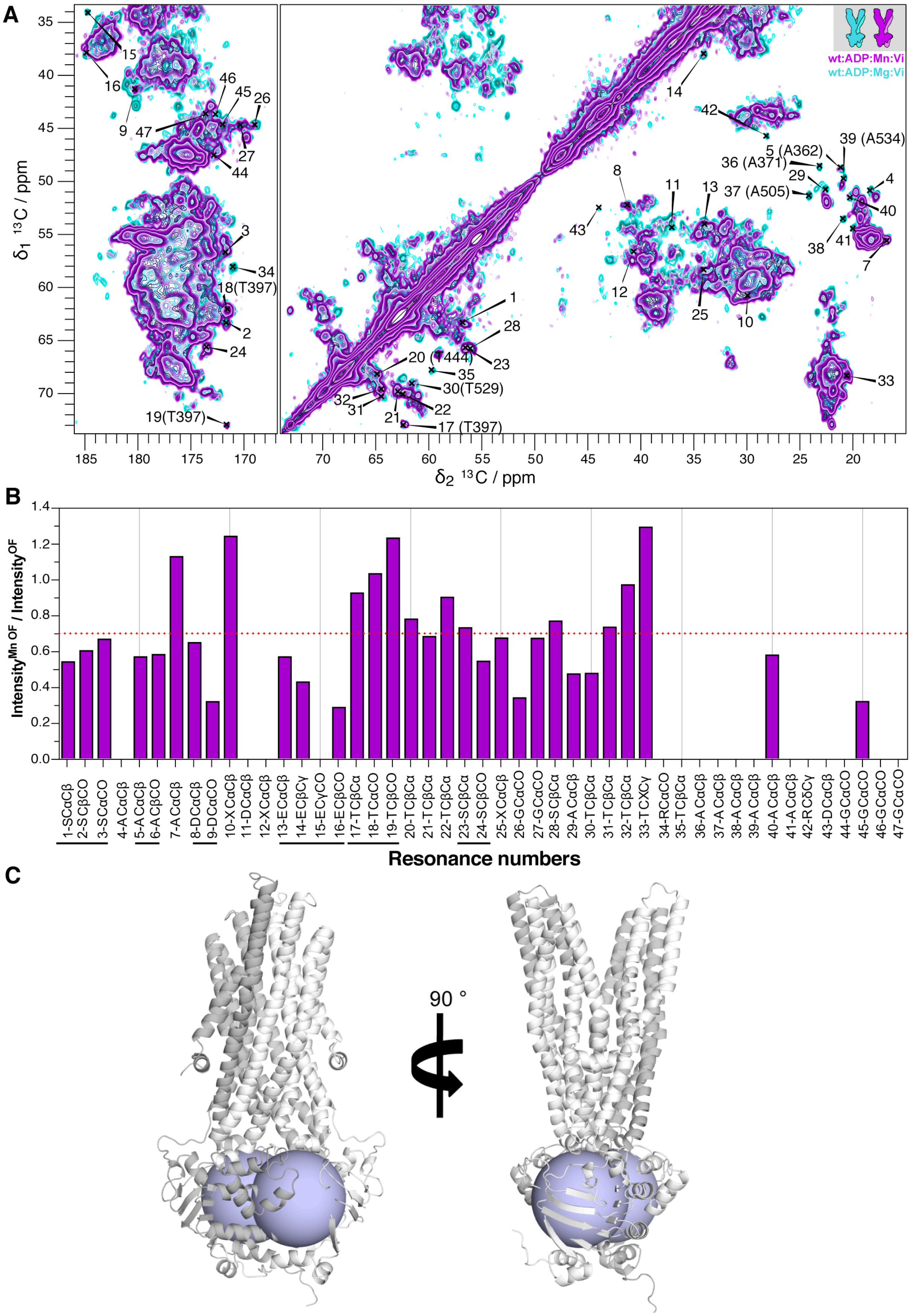

Supplementary figure 4. Extended extracts and peak analysis of wt:ADP:Mn:Vi versus wt:ADP:Mg:Vi spectra.

(A) Aliphatic and carbonyl region of 2D ^{13}C-^{13}C 20 ms DARR spectra of wt:ADP:Mn:Vi OF (purple) and wt:ADP:Mg:Vi OF (cyan) states. (*B*) Relative peak intensities (wt:ADP:Mn:Vi / wt:ADP:Mg:Vi) of the 47 peaks were calculated. Disappearing peaks in the Mn OF state are shown with zero intensity. Peaks below the red dashed line are considered to be attenuated. *(C)* The structure shown is the BmrA homology model built from Sav1866 showing only residues outside the 15 Å radius (blue sphere) around the Mn^{2+}.

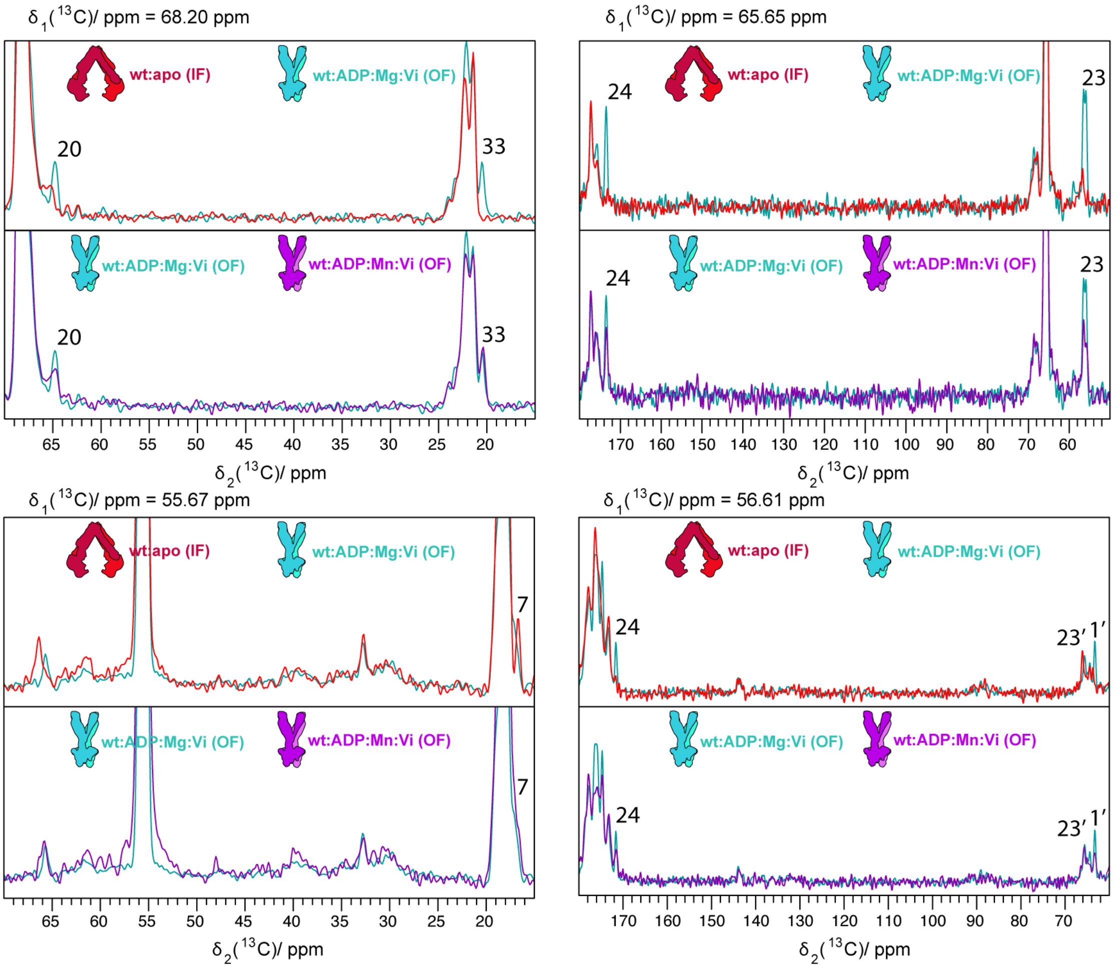

Supplementary figure 5. The conformation of the wt:ADP:Mn:Vi OF state resembles the wt:ADP:Mg:Vi OF state. Traces from 2D DARR spectra of wt:apo (red), of wt:ADP:Mg:Vi (cyan) and wt:ADP:Mn:Vi OF states (purple). Peak numbers are indicated, with corresponding peaks from the other side of the diagonal marked by '. In the upper panels, wt:apo and wt:ADP:Mg:Vi traces are overlaid to illustrate typical CSPs between the IF/OF states. In the lower panels, wt:ADP:Mg:Vi OF and wt:ADP:Mn:Vi OF traces are overlaid, to illustrate the similarity between the CSPs of these two states.

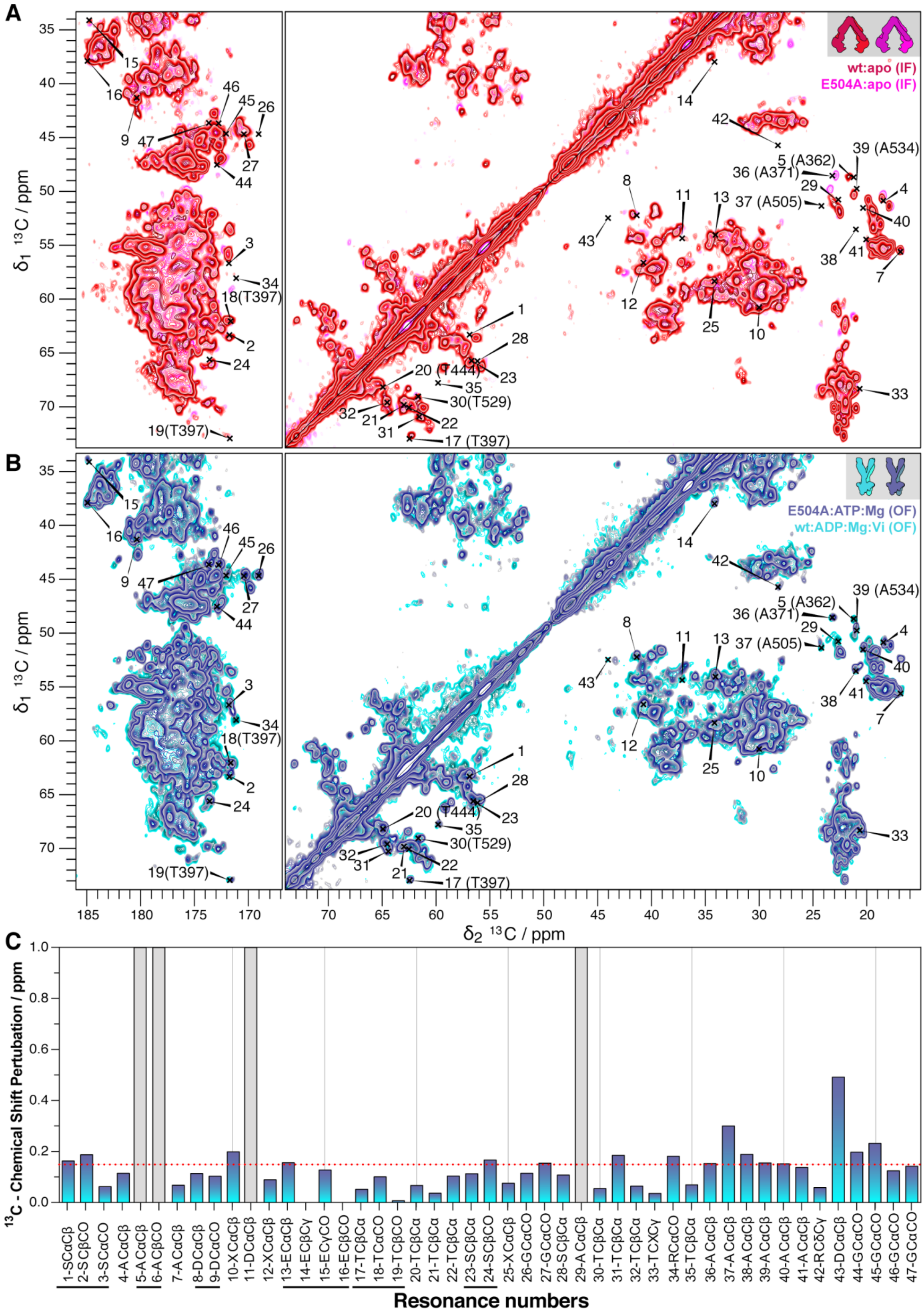

Supplementary figure 6. Extended extracts and peak analysis of E504:apo, E505:ATP:Mg, wt:apo and wt:ADP:Mg:Vi spectra. The E504A mutant in presence of ATP and magnesium makes a similar transition as the

WT protein in presence of ATP:Mg:Vi. *(A)* Aliphatic and carbonyl regions of the 2D ^{13}C-^{13}C 20 ms DARR spectra of wt:apo IF (red) and E504A:apo IF (pink) states. (*B*) Aliphatic and carbonyl regions of the 2D ^{13}C-^{13}C 20 ms DARR spectra of E504A:ATP:Mg OF (dark blue) and wt:ADP:Mg:Vi OF (cyan) states. (*C*) CSPs between E504A:ATP:Mg OF (dark blue) and wt:ADP:Mg:Vi OF (cyan) states. Peaks which are absent are represented by grey bars. The red dashed line indicates CSP higher than 0.15 ppm.

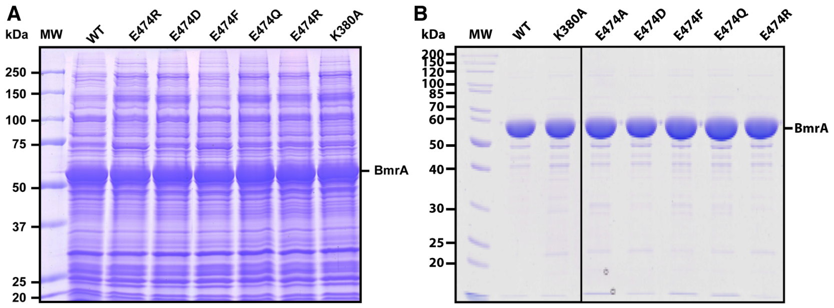

Supplementary figure 7. Expression level of BmrA in *E. coli* membrane vesicles for wild-type and mutants. (*A*) Coomassie blue-stained SDS-PAGE (10%) of membrane vesicles prepared from the C41(DE3) strain transformed with the expression vector pET23b-*bmrA* (same vector was also used for the different mutant). Lane 1 corresponds to the molecular weight markers. The position of over-expressed BmrA is indicated on the right of the gel. The amount of membrane sample deposed on each lane was around 10 µg. The expression level of the protein in the membrane is similar for each mutant. (*B*) Coomassie blue-stained SDS-PAGE (10%) of each mutant after purification, approximately 10 to 15 µg were deposed on each lane.

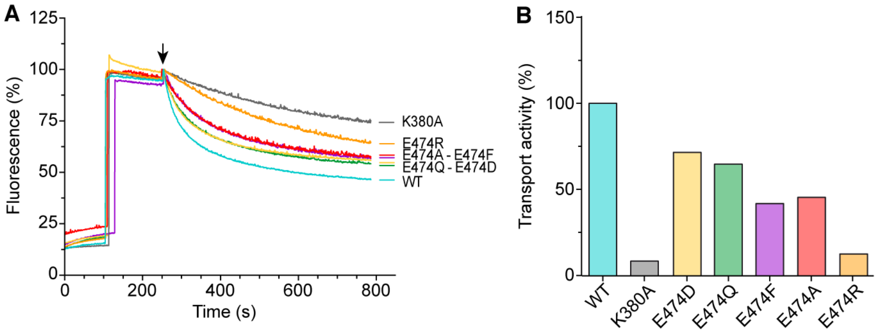

Supplementary figure 8. Transport of Hoechst 33342 by BmrA mutants measured by real-time fluorescence.
(A) ATP-dependent transport of Hoechst 33342 measured using inverted *E. coli* membrane vesicles. After addition of 2 µM Hoechst (~ 225 s), 2 mM of Mg^{2+} were added (indicated by black arrow). *(B)* Transport activities derived from initial rate fluorescence decays measured for BmrA WT and mutant forms, normalized to the rate measured for the WT form.

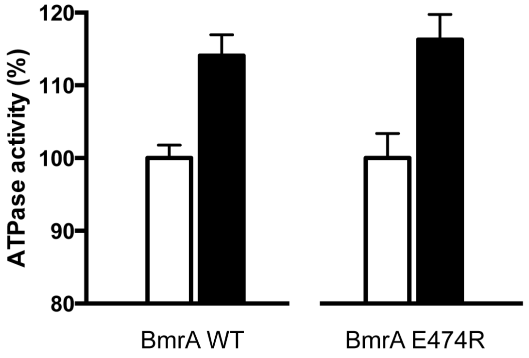

Supplementary figure 9. Drug-stimulated ATPase activities of BmrA WT and E474R. The ATPase activities of BmrA in proteoliposomes were measured in the absence (white bars) or the presence of 3.5 µM reserpine (black bars). Data were normalized to 100% basal ATPase activity for each protein. Data shows the average and standard deviation of triplicates.

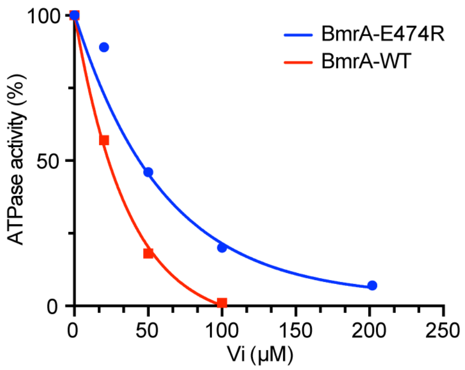

Supplementary figure 10. ATPase activities of BmrA WT and BmrA-E474R as a function of vanadate concentration. The ATPase activity was monitored using an ATP/NADH coupled assay in microplates[6]. 0.1 µg of lipid-reconstituted BmrA was mixed with 0 mM HEPES-KOH pH 8, 4 mM phosphoenolpyruvate, 60 µg/mL pyruvate kinase, 32 µg/mL lactate dehydrogenase, 10 mM $MgCl_2$, and 0.6 mM NADH. The reaction was started by the addition of 5 mM ATP, and the absorbance of NADH was followed at 340 nm during 20 min. The activities were normalized to the rate measured in the absence of vanadate.

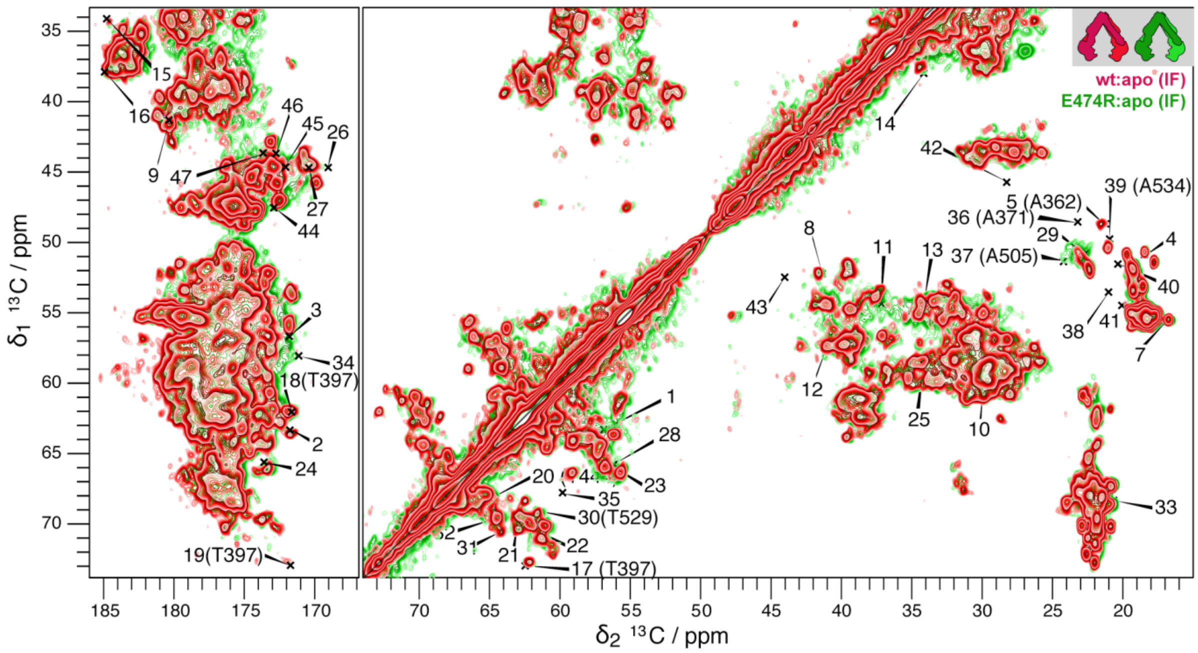

Supplementary figure 11. Extended extracts of E474R:apo versus the wt:apo spectra. The E474R mutant apo state resembles the WT apo state. Aliphatic and carbonyl regions of the 2D ^{13}C-^{13}C 20 ms DARR spectra of wt:apo (red) and E474R:apo (green) states.

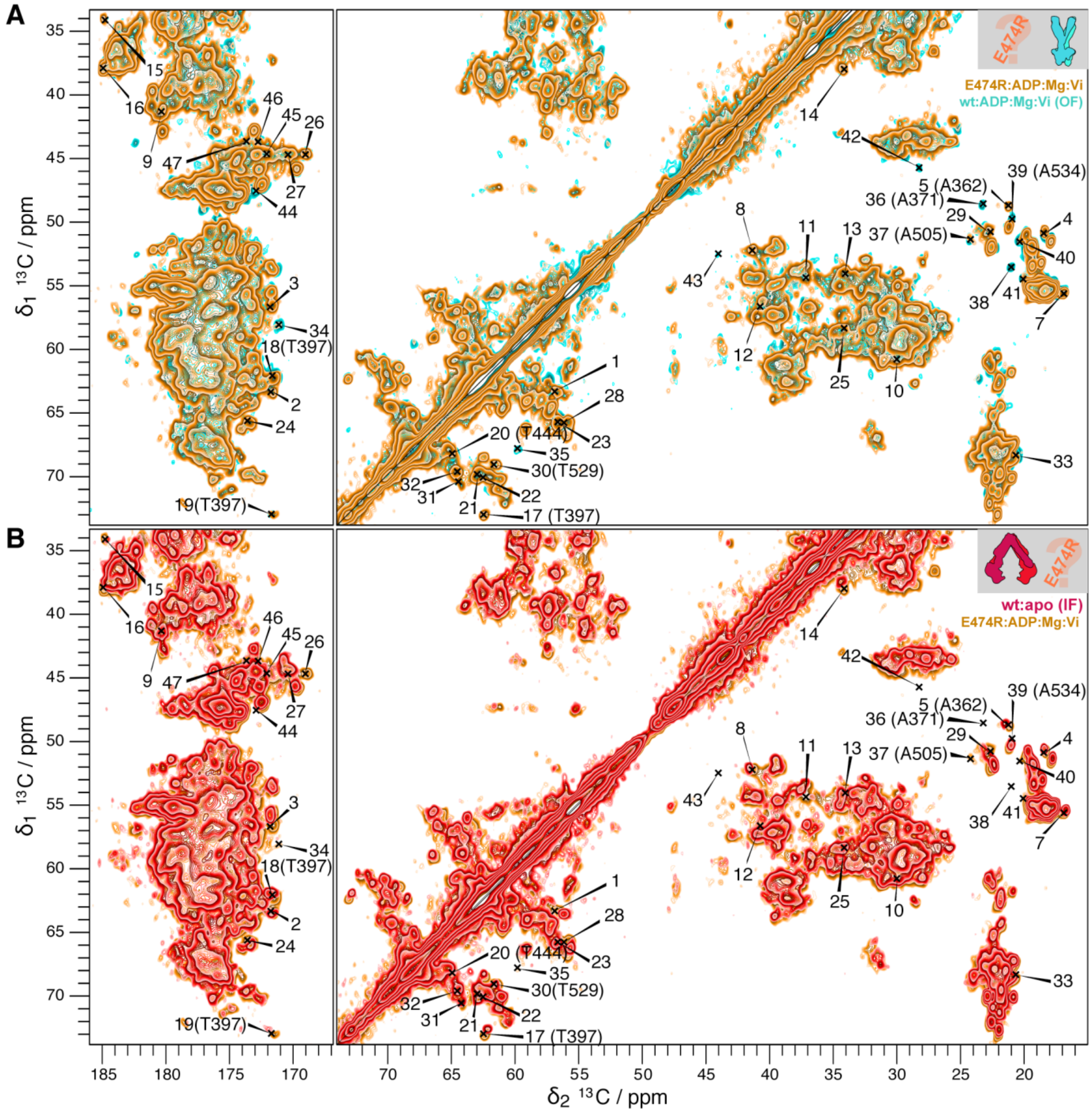

Supplementary figure 12. Extended extracts of E474R:ADP:Mg:Vi versus wt:ADP:Mg:Vi or wt:apo spectra. *(A)* Aliphatic and carbonyl regions of the 2D ^{13}C-^{13}C 20 ms DARR spectra of wt:ADP:Mg:Vi OF (cyan) and E474R:ADP:Mg:Vi (orange) states. (*B*) Aliphatic and carbonyl regions of the 2D ^{13}C-^{13}C 20 ms DARR spectra of wt:apo IF (red) and E474R:ADP:Mg:Vi (orange) states.

2D DARR	
MAS frequency/ kHz	17.5
Field/ T	18.8
Transfer I	HC-CP
^{1}H field/ kHz	70
X field/ kHz	53
Shape	Tangent ^{1}H
^{13}C carrier/ ppm	100
Transfer II	DARR
^{1}H field/ kHz	17.5
Carrier/ ppm	100
Time/ ms	20
t1 increments	1875
Sweep width (t1)/ kHz	93.75
Acquisition time (t1)/ ms	10
t2 increments	2802
Sweep width (t2)/ kHz	93.75
Acquisition time (t2)/ ms	14.94
^{1}H Spinal64 decoupling power/ kHz	90
Interscan delay/ s	2.1
Number of scans	32
Measurement time/ h	36

Supplementary Table 1. Overview of NMR parameters, all spectra were recorded the same way.

Supplementary References

1. Wiegand, T. *et al.* Monitoring ssDNA Binding to the DnaB Helicase from Helicobacter pylori by Solid-State NMR Spectroscopy. *Angew. Chem. Int. Ed.* **55,** 14164–14168 (2016).
2. Dawson, R. J. P. & Locher, K. P. Structure of a bacterial multidrug ABC transporter. *Nature* **443,** 180–185 (2006).
3. Neal, S., Nip, A. M., Zhang, H. & Wishart, D. S. Rapid and accurate calculation of protein 1H, 13C and 15N chemical shifts. *J. Biomol. NMR* **26,** 215–240 (2003).
4. Shen, Y. & Bax, A. Protein backbone chemical shifts predicted from searching a database for torsion angle and sequence homology. *J. Biomol. NMR* **38,** 289–302 (2007).
5. Williamson, M. P. Using chemical shift perturbation to characterise ligand binding. *Progr NMR Spectr* **73,** 1–16 (2013).
6. Lacabanne, D. *et al.* Gradient reconstitution of membrane proteins for solid-state NMR studies. *J. Biomol. NMR* **69,** 81–91 (2017).
